# A diffusion-weighted imaging tract-based spatial statistics study of autism spectrum disorder in preschool-aged children

**DOI:** 10.1186/s11689-019-9291-z

**Published:** 2019-12-16

**Authors:** Derek Sayre Andrews, Joshua K. Lee, Marjorie Solomon, Sally J. Rogers, David G. Amaral, Christine Wu Nordahl

**Affiliations:** The Medical Investigation of Neurodevelopmental Disorders (MIND) Institute and Department of Psychiatry and Behavioral Sciences, UC Davis School of Medicine, University of California Davis, Sacramento, CA USA

## Abstract

**Background:**

The core symptoms of autism spectrum disorder (ASD) are widely theorized to result from altered brain connectivity. Diffusion-weighted magnetic resonance imaging (DWI) has been a versatile method for investigating underlying microstructural properties of white matter (WM) in ASD. Despite phenotypic and etiological heterogeneity, DWI studies in majority male samples of older children, adolescents, and adults with ASD have largely reported findings of decreased fractional anisotropy (FA) across several commissural, projection, and association fiber tracts. However, studies in preschool-aged children (i.e., < 30–40 months) suggest individuals with ASD have increased measures of WM FA earlier in development.

**Methods:**

We analyzed 127 individuals with ASD (85♂, 42♀) and 54 typically developing (TD) controls (42♂, 26♀), aged 25.1–49.6 months. Voxel-wise effects of ASD diagnosis, sex, age, and their interaction on DWI measures of FA, mean diffusivity (MD), radial diffusivity (RD), and axial diffusivity (AD) were investigated using tract-based spatial statistics (TBSS) while controlling mean absolute and relative motion.

**Results:**

Compared to TD controls, males and females with ASD had significantly increased measures of FA in eight clusters (threshold-free cluster enhancement *p* < 0.05) that incorporated several WM tracts including regions of the genu, body, and splenium of the corpus callosum, inferior frontal-occipital fasciculi, inferior and superior longitudinal fasciculi, middle and superior cerebellar peduncles, and corticospinal tract. A diagnosis by sex interaction was observed in measures of AD across six significant clusters incorporating areas of the body, genu, and splenium of the corpus collosum. In these tracts, females with ASD showed increased AD compared to TD females, while males with ASD showed decreased AD compared to TD males.

**Conclusions:**

The current findings support growing evidence that preschool-aged children with ASD have atypical measures of WM microstructure that appear to differ in directionality from alterations observed in older individuals with the condition. To our knowledge, this study represents the largest sample of preschool-aged females with ASD to be evaluated using DWI. Microstructural differences associated with ASD largely overlapped between sexes. However, differential relationships of AD measures indicate that sex likely modulates ASD neuroanatomical phenotypes. Further longitudinal study is needed to confirm and quantify the developmental relationship of WM structure in ASD.

## Background

The core symptoms of autism spectrum disorder (ASD), i.e., deficits in social communication, social interaction and repetitive and restricted behaviors [1], are widely theorized to result from altered brain connectivity [2–5]. Magnetic resonance imaging (MRI), particularly diffusion weighted MRI (DWI), has been a versatile method for investigating underlying microstructural properties of WM in ASD in vivo. Several DWI studies have reported that individuals with ASD have atypical diffusion properties within commissural, association, and projection fiber tracts [6–8] which are likely to reflect altered neural connectivity. However, to date most of these studies have included majority male samples of older children, adolescents, and adults. In contrast, the relatively few studies that include preschool-aged children (i.e., < 50 months) suggest individuals with ASD have increased measures of WM FA earlier in life [9]. Furthermore, certain subgroups, e.g., females with ASD, remain understudied and thus associated WM neuroanatomical phenotypes in these groups remain poorly understood.

Altered neural connectivity in ASD was first proposed in terms of deficits in “long”-range connectivity combined with associated hyper “short”-range connections [2, 4]. However, a recent review of functional connectivity studies suggests that altered neural connectivity in ASD may be better understood in terms of network and/or task specific over and under connectivity [5]. In addition to functional evidence, a large body of work suggests that individuals with ASD have atypical WM structure indicative of altered structural connections. For example, significant increases in WM volumes have been observed in young children and adolescents with ASD compared to typically developing (TD) controls [10] while the corpus callosum, the largest WM fiber bundle in the brain, has been extensively studied and found to have both atypical morphology and diffusion properties in ASD [7, 8, 11–13]. Furthermore, limited postmortem evidence suggests that adults with ASD have increased numbers of thin prefrontal axons with reduced myelin density [14].

In efforts to categorize WM alterations in ASD, DWI has been particularly valuable for its ability to investigate microstructural properties of WM tracts in vivo. Most commonly, DWI studies assess the anisotropic diffusion properties characteristic of WM through tensor-based indices such as fractional anisotropy (FA) and mean diffusivity (MD) [15, 16], which have been related to several axonal properties including diameter, packing density, fiber orientation, tortuosity, membrane permeability, and myelin content [17–20]. More specific measures (i.e., axial (AD) and radial (RD) diffusivity) quantify diffusion parallel and perpendicular to the principle direction of diffusion and thus may aid in interpreting axonal properties (e.g., myelination, fiber loss) which may alter anisotropic diffusion [21].

To date, DWI studies of ASD have typically included mostly male samples of older children, adolescents and adults. For example, only 5 of 59 ASD DWI studies highlighted in a review of the literature by Ameis and Catani [6] report an ASD sample with a mean age below 5 years [22–26] and only two report samples including at least 10 ASD females [25, 27]. The large majority of DWI studies of older majority male samples which utilize tensor-based metrics have reported findings of decreased FA across several commissural, projection, and association fiber tracts, many of which have been linked to social and communicative functioning [6, 7, 11, 28–41]. However, dynamic interrelationships between brain structure and function provide a challenge in determining the underlying etiology of atypical neural connectivity in ASD based on measures gathered later in life and should be considered from a developmental perspective [42].

Accordingly, studies of early development are critical for understanding how atypical brain structure and connectivity contribute to later ASD phenotypes. Compared to studies in older individuals, relatively few DWI studies have focused on preschool-aged children (i.e., < 30-40 months). Results from these studies suggest WM neurophenotypes in ASD are characterized by increased FA earlier in development [22, 26, 43–46]. To date the large majority of MRI studies in ASD have included relatively small sample sizes (e.g., 10-20 individuals) often spanning a wide age range and multiple developmental stages (e.g., childhood, late childhood, adolescence, and adulthood). Such sampling limitations open up the possibility of averaging out and/or being underpowered to detect developmental effects. Furthermore, it is important to note that (on average) MRI samples of older individuals with ASD may differ in phenotypic severity than those in young children as nocturnal sleep protocols [47] allow for scanning of more severely affected individuals with ASD that are likely to not tolerate the nature (e.g., loud, claustrophobic) and demands (e.g., laying still for long periods of time) of MRI while awake.

Females with ASD have also been largely underrepresented in research studies. Identifying sex differences associated with ASD is critical as evidence suggests that ASD females may have distinct phenotypes from males and that factors associated with sex may modulate ASD liability (e.g., “female protective” and “male risk” models) [48]. Within TD, emerging research indicates the existence of sex differences in the structural connectome [49, 50]. Such differences represent one potential sex factor which could contribute to significant sex-by-ASD diagnosis effects that have been reported in WM structure [51–53]. Within preschool-aged samples, studies of sex differences in tensor-based metrics are limited and have included relatively small sample sizes (e.g., *n* = 7–13 ASD females) but seem to suggest similar relationships of increased FA in ASD across sexes [44, 45]. Thus, in order to determine if DWI findings in ASD are replicable in samples that more accurately represent the diversity of the autism spectrum in terms of severity and across sexes, additional research is needed.

In the current study, we sought to characterize WM diffusion properties associated with ASD in a sample of male and female preschool-aged children. We utilize DWI acquired during natural nocturnal sleep [47] to investigate measures of FA, MD, RD, and AD across whole brain WM using a voxel-wise tract-based spatial statistics (TBSS) approach [54]. We hypothesize that individuals with ASD will have significant differences in WM diffusion properties in tracts previously indicated in the condition, including the corpus callosum and superior longitudinal fasciculus. To our knowledge, our study represents the largest diffusion imaging study in terms of inclusion of preschool-aged females with ASD. Based on prior DWI findings from our group reporting significant sex differences in TD [55] and diagnosis-by-sex interaction effects in ASD [52], we anticipate both a significant main effect of sex and diagnosis-by-sex interactions in diffusion measures.

## Methods

### Participants

We analyzed a cross sectional sample of 127 individuals with ASD (85♂, 42♀) and 54 typically developing (TD) controls (42♂, 26♀), ages 25.1–49.6 months (Table 1). Participants were enrolled in either the ongoing UC Davis Medical Interventions in Neurodevelopmental Disorders (MIND) Institute longitudinal Autism Phenome Project (APP) or Girls with Autism: Imaging of Neurodevelopment (GAIN) studies. The design of these studies involves enrolling and conducting baseline MRI in children at 24–42 months of age and then imaging at annual intervals for two additional time points. The current cross sectional study sample included all individuals in the APP/GAIN cohorts below the age of 50 months who had successfully completed structural, diffusion-weighted, and phase-mapping MRI scans post an MRI scanner upgrade in August 2009. Previous DWI studies that have utilized subgroups of the currently described sample included data acquired both prior to and after this upgrade [52, 55]. In cases where participants had successfully completed scanning at more than one time point prior to 50 months, data from their first (i.e., youngest) available time point was always used.

**Table 1:** Participant demographics

	Full Sample		Males		Females	
	ASD (*n* = 127)	TD (*n* = 54)	ASD (*n* = 85)	TD (*n* = 28)	ASD (*n* = 42)	TD (*n* = 26)
Age (months)	38.8 (5.6) [25.9–49.6]^*^	36.4 (6.8) [25.1–49.3]^*^	38.8 (5.8) [25.9–49.6]^*^	35.2 (6.6) [25.1–45.0]^*^	38.8 (5.3) [29.5–49.0]	37.7 (6.9) [27.9–49.3]
DQ	65.39 (21.76) [23–113]^**^	104.95 (11.97) [73–129]^**^	63.33 (20.90) [29–113]^**^	102.69 (11.33) [82–123]^**^	69.57 (23.10) [23–113]^**^	107.39 (12.37) [73–129]^**^
ADOS	7.47 (1.82) [4–10]	-	7.48 (1.79) [4–10]	-	7.45 (1.88) [4–10]	-
ADI SOC	17.00 (4.17) [7–26]	-	16.69 (4.09) [8–25]	-	17.64 (4.32) [7–26]	-
ADI BEH	5.51 (2.12) [0–12]	-	5.71 (2.16) [0–12]	-	5.11 (2.01) [1–11]	-
ADI COM	10.84 (3.2) [1–21]	-	10.64 (2.97) [4–21]	-	11.23 (3.66) [1–18]	-
RMS Absolute (mm)	0.34 (0.14)	0.34 (0.12)	0.35 (0.15)	0.32 (0.11)	0.33 (0.12)	0.37 (0.13)
RMS Relative (mm)	0.37 (0.17)	0.34 (0.08)	0.37 (0.18)	0.33 (0.08)	0.37 (0.15)	0.36 (0.08)

Note: Participant demographics for full sample, male and female subgroups. Mean (standard deviation) [range]. *ASD* autism spectrum disorder, *TD* typical development, *DQ* Mullen developmental quotient, *ADOS* autism diagnostic observation schedule calibrated severity score, *ADI* autism diagnostic interview, *SOC* social, *COM* communication, *BEH* repetitive behavior sub scales, *RMS* root-mean-square absolute and relative motion. TD controls were not assessed using ADOS or ADI

^*^Significant difference *p* < 0.05

^**^p < 0.001

All participants were required to be native English speakers, ambulatory, have no contraindications for MRI, no suspected vision or hearing problems or known genetic disorders or neurological conditions. An ASD diagnosis was confirmed at study entry by trained clinical psychologists using the Autism Diagnostic Observation Schedule-Generic (ADOS-G) [56] or ADOS-2 [57], the Autism Diagnostic Interview-Revised (ADI-R) [58] and DSM-IV-TR criteria [1]. Based on their scores on these measures, participants were included according to criteria for young children with ASD established by the Collaborative Programs of Excellence in Autism network. As specified by these criteria, all ASD participants met ADOS-2 cutoff scores for either autism or ASD. In addition, they exceeded ADI-R cutoff scores for autism on either the social or communication subscale and were within two points of this criterion on the other subscale. ADOS-calibrated severity scores were calculated to allow comparison of autism severity across participants tested with different ADOS modules [59]. At Time 1, TD individuals were screened for autism traits using the Social Communication Questionnaire (SCQ) (i.e., scores below 11) [60] and were required to have no first-degree relatives with an ASD diagnosis.

The Mullen Scales of Early Learning (MSEL) [61] was used to assess developmental quotient (DQ) during participants first visit (Time 1). TD children were excluded if they did not fall within two standard deviations on the MSEL. MRI data from the second visit (Time 2) for 17 participants’ (*n* = 11 ASD♂, 4 TD♂, 1 ASD♀, 1 TD♀) was used due to quality issues with or failure to acquire their Time 1 MRI data. For these 17 participants, we report MSEL, ADOS, and ADI scores from their first visit. All aspects of the study protocol were approved by the University of California, Davis Institutional Review Board, and informed consent was obtained from the parent or guardian of each participant.

### Image acquisition

All MRI scanning was performed at the Imaging Research Center, UC Davis, Sacramento, during natural nocturnal sleep without sedation [47] from October 2009 to July 2018, using a 3-T Siemens Magnetom Trio MR system (Erlangen, Germany) with an 8-channel head coil. High-resolution T1 images were acquired using an MPRAGE sequence (1 mm^3^ resolution, TR = 2170 ms, TE = 4.86 ms, TI = 1100 ms, FA = 7°, 192 slices, 256 × 256 × 192 mm FOV). Diffusion-weighted images (DWI) were acquired in 30 independent directions along with five interleaved non-diffusion weighted (*b* = 0) images (1.9 mm^3^ resolution, TR = 8500 ms, TE = 81 ms, *b* = 700, echo spacing = 0.69 ms, GRAPPA iPAT factor = 2, 72 slices, 243 × 243 × 137 mm FOV). An accompanying phase map image was acquired using the same shim as the DWI sequence to correct for field inhomogeneities (4 mm^3^ resolution, TR = 1000 ms, TE = 3.60/6.06 ms, FA = 90°, 48 slices, 256 × 256 × 230 mm FOV).

### Diffusion-weighted image preprocessing

Diffusion-weighted images were preprocessed using the MRtrix3 package (www.mrtrix.org) which utilizes elements of the FSL ([62]; fsl.fmrib.ox.ac.uk) diffusion toolbox (e.g., *“eddy”* [63]). Preprocessing steps included (1) image denoising according to a principle component analysis-based method [64, 65], (2) Gibbs ringing artifact reduction [66], (3) correction for distortion due to eddy currents and between volume movements using FSL’s *eddy* tool [﻿63﻿] with the options to (4) replace slices with average intensity at least four standard deviations lower than the expected intensity with an interpolated Gaussian process prediction [67], and perform (5) within volume (i.e., slice to volume) motion correction [68], the latter of which utilizes the NIVIDA CUDA parallel computing platform (developer.nvidia.com/cuda-zone). (6) Individual field map images were then calculated and used to correct for field distortions while simultaneously registering the diffusion images to their corresponding T1-weighted image using FSL *epi_reg* [69–71]. (7) Lastly, all preprocessed volumes were visually screened by the first author to insure quality of between volume registration and to identify potential image misorientation, slice dropout, and distortion effecting WM regions.

### Head motion

Image artifacts associated with head motion are a significant confound in ASD research. Head motion has shown to be increased in ASD [72] and to significantly impact DWI results [73]. Accordingly, in addition to utilizing a noctoral sleep protocol [47] and state of the art within volume motion correction [68], we quantified head motion using the root-mean-square (RMS) displacement of both the mean absolute intervolume displacement with respect to the first image of each acquisition and the mean relative intervolume displacement between each preceding image in the sequence. Participants with a mean absolute RMS displacement greater than 1.0 mm (*n* = 4 ASD♂, 0 TD♂, 2 ASD♀, 1 TD♀) were excluded from further analysis and are not described in this study. For all other participants, mean absolute and relative RMS displacement across volumes were included as covariates in all further analyses.

### Diffusion tensor modeling and tract-based spatial statistics

Diffusion was modeled by the fitting of a tensor at each voxel using FSL’s diffusion toolbox. Each tensor can be defined by its three principle Eigen vectors (i.e., *λ*_1_, *λ*_2_, *λ*_3_). Tensor maps were used to calculate corresponding maps of fractional anisotropy (FA; $\sqrt{\frac{{\left({\lambda}_1-{\lambda}_2\right)}^2+{\left({\lambda}_2-{\lambda}_3\right)}^2+{\left({\lambda}_1-{\lambda}_3\right)}^2}{2\left({\lambda}_1^2+{\lambda}_2^2+{\lambda}_3^2\right)}}$), mean diffusivity (MD; (*λ*_1_ + *λ*_2_ + *λ*_3_)/3), radial diffusivity (RD; (*λ*_2_ + *λ*_3_)/2), and axial diffusivity (AD; *λ*_1_).

Whole-brain voxel-wise statistical analysis of FA, MD, RD, and AD maps was conducted using tract-based spatial statistics (TBSS) [54]. First, BET brain extraction [74] was performed on each FA image and end slices zeroed to remove likely outliers from the tensor fitting. A study-specific template was then derived by registering each individual’s FA image to all other FA images (i.e., *tbss_2_reg -n*). The image found to be most representative of the sample (i.e., target image) was then affine-aligned into MNI152 standard space. All FA images were then registered to MNI152 by combining the nonlinear transform to the target image with the affine transformation of the target to MNI152 space. A mean FA image of all participants was then used to derive a white matter “skeleton” which was thresholded to include FA values > 0.2. This resulting white matter skeleton was then used as a binary mask on which individual’s measures of FA, MD, RD, and AD were separately projected and subsequently exported for voxel-wise statistical analysis.

### Statistical analyses

Non-parametric statistical inference of voxel-wise TBSS-skeletonized measures of FA, MD, RD, and AD were estimated by regression of a general linear model using FSL’s “*randomise*” [75]. Diagnostic group and sex were included as categorical factors with age in months, mean absolute, and relative movement as continuous covariates:

$$ {Y}_i={\beta}_0+{\beta}_1\mathrm{Diagnosis}+{\beta}_2\mathrm{Sex}+{\beta}_3\mathrm{Age}+{\beta}_4\mathrm{absMove}+{\beta}_5\mathrm{relMove}+{\varepsilon}_i $$

where *ε*_*i*_ is the residual error at voxel *i*. Diagnosis-by-sex (*β*_1_Diagnosis ∗ *β*_2_Sex), diagnosis-by-age (*β*_1_Diagnosis ∗ *β*_3_Age), and sex-by-age (*β*_2_Sex ∗ *β*_3_Age), interaction effects were tested by adding these terms separately to the above model. Diagnosis-by-sex-by-age (*β*_1_Diagnosis ∗ *β*_2_Sex ∗ *β*_3_Age) interaction effects were tested for by adding this and the lower order two-way interaction terms to the above model. Statistical thresholding and correction for multiple comparisons was conducted via a threshold-free cluster enhancement (TFCE) [76] permutation (*n* = 10,000) paradigm to identify significant (*p* < 0.05) effects of diagnosis (*β*_1_), sex (*β*_2_), age (*β*_3_), and the above interaction terms for each DWI measure.

## Results

### Participant demographics

Across the entire sample (i.e., males and females), individuals with ASD were found to be significantly younger than TD controls (*t* = 2.45, *p* = 0.01). This effect was driven primarily by a significant difference in age between ASD and TD males (*t* = 2.72, *p* = 0.008) which was not observed between ASD and TD females (*t* = 2.45, *p* = 0.45). Across diagnostic groups, males did not significantly differ in age from females (*t* = − 0.53, *p* = 0.59). As expected, individuals with ASD had significantly lower MSEL DQ scores than TD participants (*t* = − 12.55, *p* = <0.001). Across diagnostic groups, no significant difference in MSEL DQ was found between males and females (*t* = − 1.52, *p* = 0.12). No significant differences in ADOS severity scores, ADI social, behavior or communication measures were observed between males and females with ASD diagnoses (*p* > 0.05). No significant differences between diagnostic groups or sexes were observed for mean absolute or mean relative RMS motion parameters (*p* > 0.05). See Table 1 for participant demographics.

### Diagnostic group differences in white matter diffusion properties

**Fig. 1:**
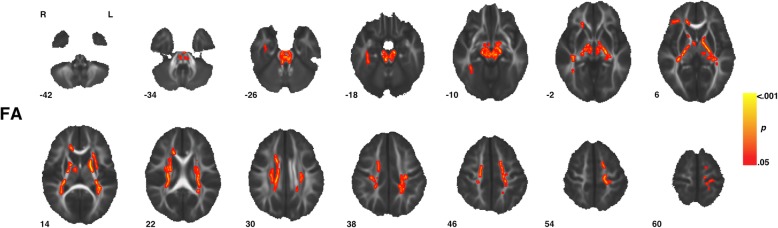
Regions of increased fractional anisotropy in ASD. Individuals with ASD diagnoses showed significantly (TFCE *p* < 0.05) increased measures of fractional anisotropy (FA) across eight clusters (Table 2) highlighted above. Indicated white matter tracts include regions of the corpus callosum, corona radiata, and inferior and superior longitudinal fasciculi as well as the middle and superior cerebellar peduncles and corticospinal tract. Images are presented in R/L radiological convention with MNI *z* coordinates in millimeter. Skeletonized statistical overlays have been “inflated” for display

**Fig. 2:**
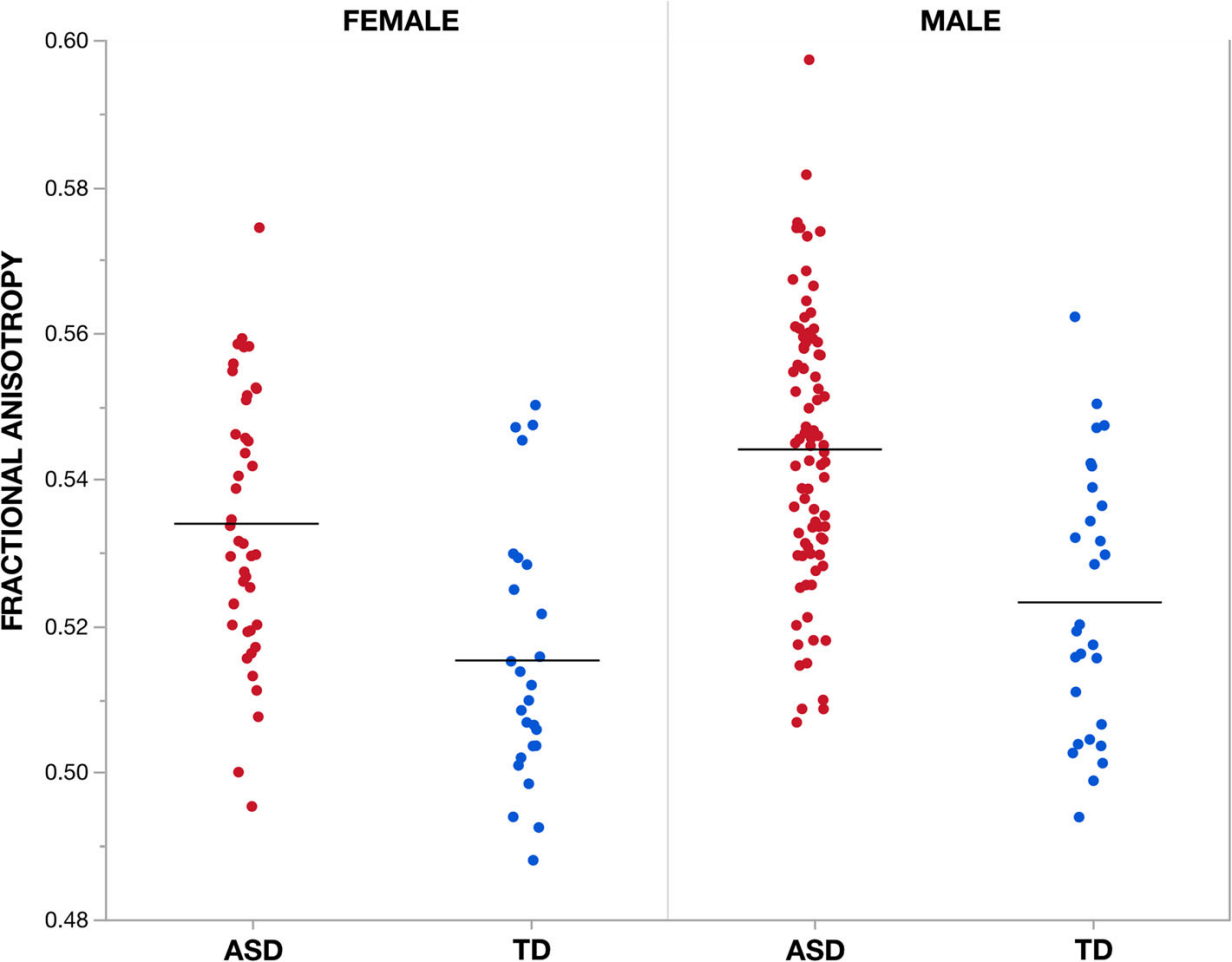
Effect of group on fractional anisotropy measures across individuals. Mean fractional anisotropy (FA) measures within the largest cluster (i.e., cluster 1) showing a significant (TFCE *p* < 0.05) effect of group (ASD > TD) are plotted for each individual according to group and sex. Cluster 1 incorporates bilateral regions of the middle and superior cerebellar peduncles, corticospinal tract, cerebral peduncle, and internal capsule as well as left corona radiata, thalamic radiation, external capsule, fornix, superior longitudinal fasciculus and fronto-occipital fasciculus. Of note, both males and females with ASD diagnoses show increased FA compared to TD males and females

Voxel-wise analysis showed individuals with ASD compared to TD controls had significantly (TFCE *p* < 0.05) increased FA in eight clusters that incorporated several white matter tracts including regions of the corpus callosum, corona radiata, and inferior and superior longitudinal fasciculi as well as the middle and superior cerebellar peduncles, and corticospinal tract (Fig. 1, Table 2). Within all eight clusters, increased FA in ASD was observed across sexes, i.e., increased FA in ASD was not sex-specific (Fig. 2). No clusters exhibiting significant (TFCE *p* < 0.05) between group differences were observed for measures of MD, RD, or AD.

**Table 2:** Clusters with significant effect of group and group by sex interaction

Effect	Feature	Cluster ID	Tract(s)	*p*	*t*_max_	voxels	*X* (mm)	*Y* (mm)	*Z* (mm)
Diagnosis	FA	1	R/L middle/superior cerebellar peduncle, corticospinal tract, cerebral peduncle, anterior/posterior internal capsule, L anterior/superior/posterior corona radiata, posterior thalamic radiation, external capsule, fornix, superior longitudinal fasciculus, superior, fronto-occipital fasciculus	0.013	4.57	5748	5	− 30	− 18
		2	R anterior/posterior internal capsule, superior/posterior corona radiata, posterior thalamic radiation, inferior longitudinal fasciculus, inferior frontal-occipital fasciculus, external capsule	0.026	4	1416	30	− 31	12
		3	Genu/body/splenium corpus callosum, R anterior/superior/posterior corona radiata	0.015	5.17	1037	17	2	33
		4	R inferior longitudinal fasciculus, inferior fronto-occipital fasciculus	0.038	4.02	399	36	− 34	− 18
		5	R anterior corona radiata	0.045	3.7	79	38	37	6
		6	R anterior corona radiata	0.048	3.35	48	19	29	− 5
		7	R anterior limb internal capsule	0.047	3.66	28	16	10	9
		8	R forceps major, posterior corona radiata	0.05	3.29	6	35	− 63	19
Diagnosis-by-sex	AD	1	Genu/body corpus callosum, R anterior/superior corona radiata	0.036	3.61	532	16	2	32
		2	R anterior/superior corona radiata, external capsule	0.04	3.51	445	25	28	19
		3	Body/splenium corpus callosum, R posterior corona radiata	0.038	4.14	263	16	− 26	30
		4	Genu/body corpus callosum	0.042	3.39	229	1	12	20
		5	R anterior corona radiata	0.047	2.96	80	30	18	30
		6	Body corpus callosum	0.045	3.57	45	− 1	− 4	24

Note: Clusters of significant group differences (ASD > TD) and diagnosis-by-sex interaction effects. Fractional anisotropy (FA), axial diffusivity (AD), tracts identified according to the *MRI Atlas of Human White Matter* [77], L (left), R (right), *p* indicates the threshold-free cluster enhancement-corrected *p* value for the cluster, *t*_max_ indicates maximum *t* statistic within the cluster at X Y Z MNI coordinates in millimeters

### Main effects of age and sex in white matter diffusion properties

**Fig. 3:**
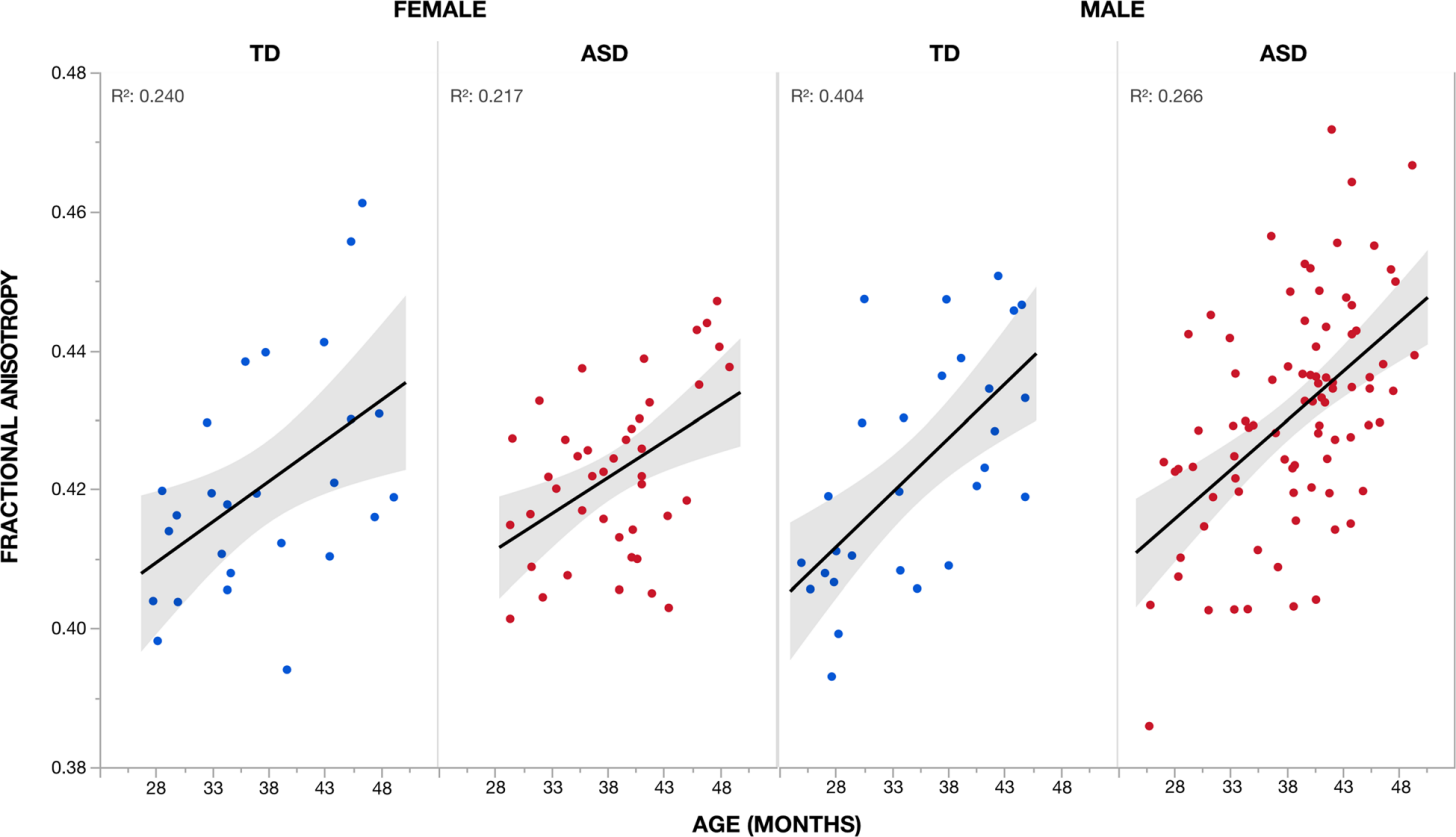
Effect of age on fractional anisotropy across individuals. Mean fractional anisotropy (FA) measures for the cluster (Additional file 3: Table S1) showing a significant (TFCE *p* < 0.05) positive effect of age are plotted for each individual according to group and sex. This cluster incorporated a majority of all white matter tracts (Additional file 1: Figure S1). Increases in FA with age were observed across both groups (i.e., ASD and TD) and sexes (i.e., male and female). Coefficients of determination (*R*^2^) for goodness of fit are provided. Shaded region indicates 95% confidence interval

Voxel-wise analysis showed a significant (TFCE *p* < 0.05) main effect of age for all children (i.e., across both diagnostic groups and sexes) in all four diffusion measures in expansive overlapping clusters that incorporated a majority of all white matter tracts (Additional file 1: Figure S1, Additional file 3: Table S1). Increased FA with age was accompanied by decreased MD, RD, and AD in these clusters. Similar trajectories of increased FA with age were observed across sexes and groups (Fig. 3).

Furthermore, across diagnostic groups, males were found to have significantly (TFCE *p* < 0.05) increased measures of FA compared to females across six clusters that incorporated a majority of all white matter tracts. Overlapping significant decreases in MD and RD were observed in several of these tracts, but were absent in some posterior tracts including the posterior thalamic radiation, forceps major, and retrolenticular part of the internal capsule (Additional file 2: Figure S2, Additional file 3: Table S2). No clusters showing significant effects of sex were found for measures of AD.

### Interaction effects between diagnosis, sex, and age in white matter diffusion properties

**Fig. 4:**
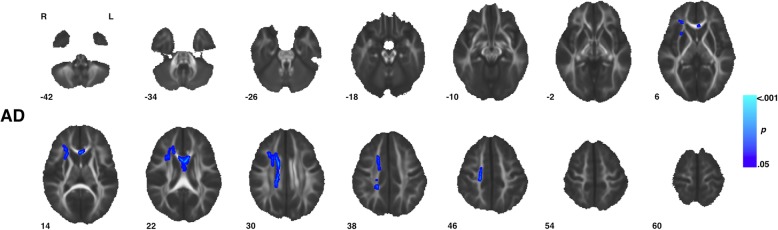
Regions with group by sex interaction in axial diffusivity. Clusters (Table 2) showing a significant (TFCE *p* < 0.05) group by sex interaction effect in measures of axial diffusivity are highlighted. In total, six clusters incorporated areas of the body, genu, and splenium of the corpus collosum as well as areas of the right corona radiata and external capsule. Within these regions, ASD males showed decreased AD compared to TD males while ASD females showed increased AD compared to TD females (Fig. 7). Images are presented in R/L radiological convention with MNI *z* coordinates in millimeters. Skeletonized statistical overlays have been “inflated” for display

**Fig. 5:**
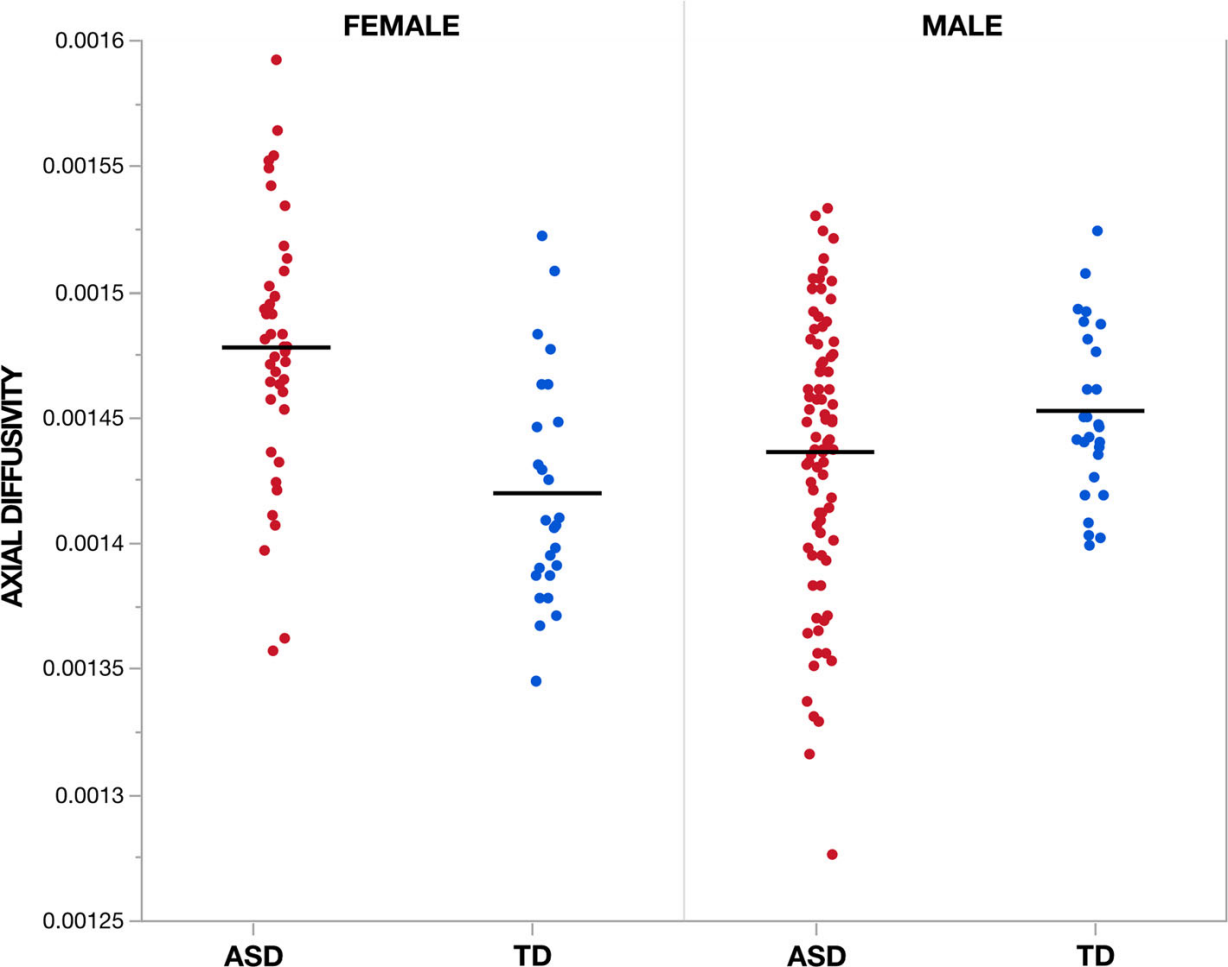
Group by sex interaction effects in axial diffusivity across individuals. Individual’s mean axial diffusivity (AD) measures are plotted according to group and sex for the largest cluster (1) for which a significant (TFCE *p* < 0.05) group by sex interaction effect was observed. Cluster 1 incorporates regions of the genu and body of the corpus callosum as well as the right anterior and superior corona radiata. Across all six clusters, ASD males showed decreased AD compared to TD males while ASD females showed increased AD compared to TD females. Units for measures of AD are given in mm^2^/s

Voxel-wise analysis showed no significant (TFCE *p* < 0.05) diagnosis-by-age, sex-by-age, or diagnosis-by-sex-by-age interaction effects across all four diffusion measures. However, significant diagnosis-by-sex interactions were observed in measures of AD across six clusters incorporating areas of the body, genu, and splenium of the corpus collosum as well as areas of the right corona radiata and external capsule (Fig. 4, Table 2). Within these regions ASD males showed decreased AD compared to TD males while ASD females showed increased AD compared to TD females (Fig. 5). No significant (TFCE *p* < 0.05) diagnosis-by-sex interaction effects were observed for measures of FA, MD, or RD.

## Discussion

Our aim was to characterize WM structural properties associated with ASD in preschool-aged children using a whole-brain, voxel-wise DWI approach. We found that individuals with ASD had significantly increased measures of FA compared to TD controls within several commissural, association, and projection WM tracts. While both males and females with ASD demonstrated increased FA, significant sex-by-diagnosis interactions in measures of AD indicate that sex differences modulate WM neuroanatomical phenotypes in ASD. Caution must be taken in interpreting altered anisotropic diffusion properties as directly reflecting increased or decreased connectivity in ASD [78]. However, these findings support growing evidence that young children with ASD have atypical measures of WM microstructure [9, 22, 26, 43–46] that may contribute to core ASD symptomatology and differ in directionality from alterations observed in older children, adolescences and adults with the condition [6, 7, 11, 28–34, 36–41].

Of the WM tracts identified as having increased measures of FA, the corpus callosum is the most widely studied and implicated in ASD [7, 8, 13]. This tract provides extensive long-range connections in the brain and has been implicated in social and communicative functioning [79]. Within ASD, individuals have been shown to have smaller callosal volumes [12, 13, 52] and reduced interhemispheric functional connectivity suggestive of deficits in commissural tract integrity [80]. We also identified increased FA within the inferior longitudinal and inferior frontal-occipital fasiculi. Both of these tracts have been indicated in prior DWI studies of ASD [6, 7, 33–35, 37] and have shown to be important in the recognition of emotional facial expressions [81]. Of note, the largest cluster of increased FA in the current study incorporated the middle and superior cerebellar peduncles. While classically associated with motor coordination [82], recent evidence suggests that the cerebellum plays a critical role in the adaptive control of cortical processing [83] and has been linked to the establishment of normative social behaviors in preclinical models of ASD [84]. Postmortem studies of ASD have noted atypical Purkinje cell density in the cerebellum [85, 86] indicating early disruption of cerebellar development in the condition. Recently reported atypical expression of oligodendrocyte-specific genes in the cerebellum of individuals with ASD highlights one potential pathway towards altered cerebellar development and myelination in the condition [87]. Collectively, the current observation of atypical measures of WM microstructure and/or fiber orientation within these tracts appears likely to reflect atypical neural connectivity associated with ASD.

These findings support a growing body of evidence that indicates young children with ASD have increased FA compared to TD controls [9, 22, 26, 43–46]. Given ASD likely manifests prenatally [88] and is first clinically diagnosable around 2 years of age, early-life measures of brain structure and connectivity not only are critical to understanding the biological basis of autism but also need to be considered from a developmental perspective [42]. To date a large majority of DWI studies have reported atypical measures of WM microstructure in older children, adolescents, and adults with ASD in the form of decreased FA, often accompanied by increased MD, in WM tracts implicated in social functioning [6, 7, 11, 28–41]. Based on previous findings, the transition from increased FA in younger children with ASD to observed decreases later in life appears to manifest sometime between 30 and 40 months of age [9, 44], suggesting WM undergoes an atypical developmental trajectory in ASD.

Our study focused on a cross sectional sample and is thus not able to directly address hypotheses relating longitudinal changes. However, the age range of the current cohort (~ 20–50 months) does capture the period of development when increased FA would be hypothesized to transition to decreased FA in the condition. Within our cohort, across both groups and sexes, we observed increased FA and decreased MD, RD, and AD with age across a large majority of all WM tracts. We did not observe significant diagnosis-by-age effects. Thus, our findings do not suggest a differential developmental trajectory in measures of diffusion properties associated with ASD across the age range of our sample (i.e., ~ 20–50 months). This is in contrast to two studies that have tracked DWI measures in ASD longitudinally prior to 50 months of age, albeit in relatively small samples, that suggest that early increases in FA later develop into decreased FA in ASD [44, 45]. Accordingly, the current study highlights the need for additional longitudinal investigations of WM structure to fully categorize the developmental relationships of DWI measures in ASD across early development and into middle childhood, adolescence, and adulthood.

To our knowledge, this study includes the largest DWI sample of preschool-aged females with ASD. This is important as females are largely underrepresented in ASD research and may have differences in both behavioral and neuroanatomical phenotypes from males with the condition [48]. Across diagnostic groups, we observed a significant main effect of sex characterized by increased FA and accompanying decreased MD and RD in males compared to females across a majority of all WM tracts. The global nature of these sex effects suggests a mediating role of differential sexual processes (e.g., steroid hormones) during early development on WM microstructure [89]. Findings of increased FA in males have been reported by others [90, 91] as well as by a previous study that included a portion of the TD control participants currently described [55]. Within the current study, both males and females with ASD showed similar relationships of increased FA compared to TD controls in the tracts described above. However, we did observe a significant diagnosis-by-sex interaction in measures of AD mainly within the genu and body of the corpus callosum as well as anterior and superior regions of the corona radiata. Within these clusters, females with ASD showed increased AD compared to TD females, while males with ASD had decreased AD relative to TD males. Differences in AD between ASD and TD were also larger in females than males. This result is similar to a prior study from our group that identified increased AD, RD, and MD in the corpus callosum of females with ASD but not males compared to TD controls [52]. As AD quantifies the principle direction of diffusion within a voxel, of the currently studied measures of diffusion anisotropy, AD is likely to be particularly sensitive to overall fiber orientation. Thus, the current finding may reflect an interaction of TD sex differences in the structural organization of WM connections [49, 50] and sex differences associated with ASD neuroanatomical phenotypes [52]

## Conclusion

Findings of increased FA in preschool-aged children with ASD suggest that altered WM structural properties are evident in ASD at an age when current diagnostic assessment of the condition is first possible and that these differences are likely to be reflective of atypical neural connectivity. Similar differences in WM microstructure were observed in both ASD males and females, although differential relationships of measures of AD between sexes indicate a mediating role of sex in WM microstructure and/or fiber orientation in the condition. We did not observe evidence of different age-related effects in DWI measures between groups within our cross sectional sample. This study represents a primary analysis to characterize WM structural properties in a subsample of children under 50 months of age. A follow up longitudinal study will be required to confirm and quantify the developmental relationship of WM structure in ASD and across sexes.

## Supplementary information

**Additional file 1: Figure S1.** Effects of Age on Measures of Diffusion. Clusters (Additional file 3: Table S1) of significantly (TFCE *p*<0.05) increased fractional anisotropy (FA) and decreased mean diffusivity (MD), radial diffusivity (RD), and axial diffusivity (AD) with age are highlighted. Images are presented in R/L radiological convention with MNI *z* coordinates in mm. Skeletonized statistical overlays have been ‘inflated’ for display.

**Additional file 2: Figure S2.** Effects of Sex on Measures of Diffusion. Clusters (Additional file 3: Table S2) showing significantly (TFCE *p*<0.05) increased fractional anisotropy (FA) and decreased mean diffusivity (MD) and radial diffusivity (RD) in males compared to females across diagnostic groups are highlighted. Images are presented in R/L radiological convention with MNI *z* coordinates in mm. Skeletonized statistical overlays have been ‘inflated’ for display.

**Additional file 3: Table S1.** Clusters with Significant Effect of Age. **Table S2.** Clusters with Significant Effect of Sex.

## Data Availability

Data described in the current study is available from the corresponding author on reasonable request.
