# Supplementary figures and images for: A diffusion-weighted imaging tract-based spatial statistics study of autism spectrum disorder in preschool-aged children
Source: J Neurodev Disord. 2019 Dec 16;11:32. doi: 10.1186/s11689-019-9291-z (PMC6913008; doi:10.1186/s11689-019-9291-z)

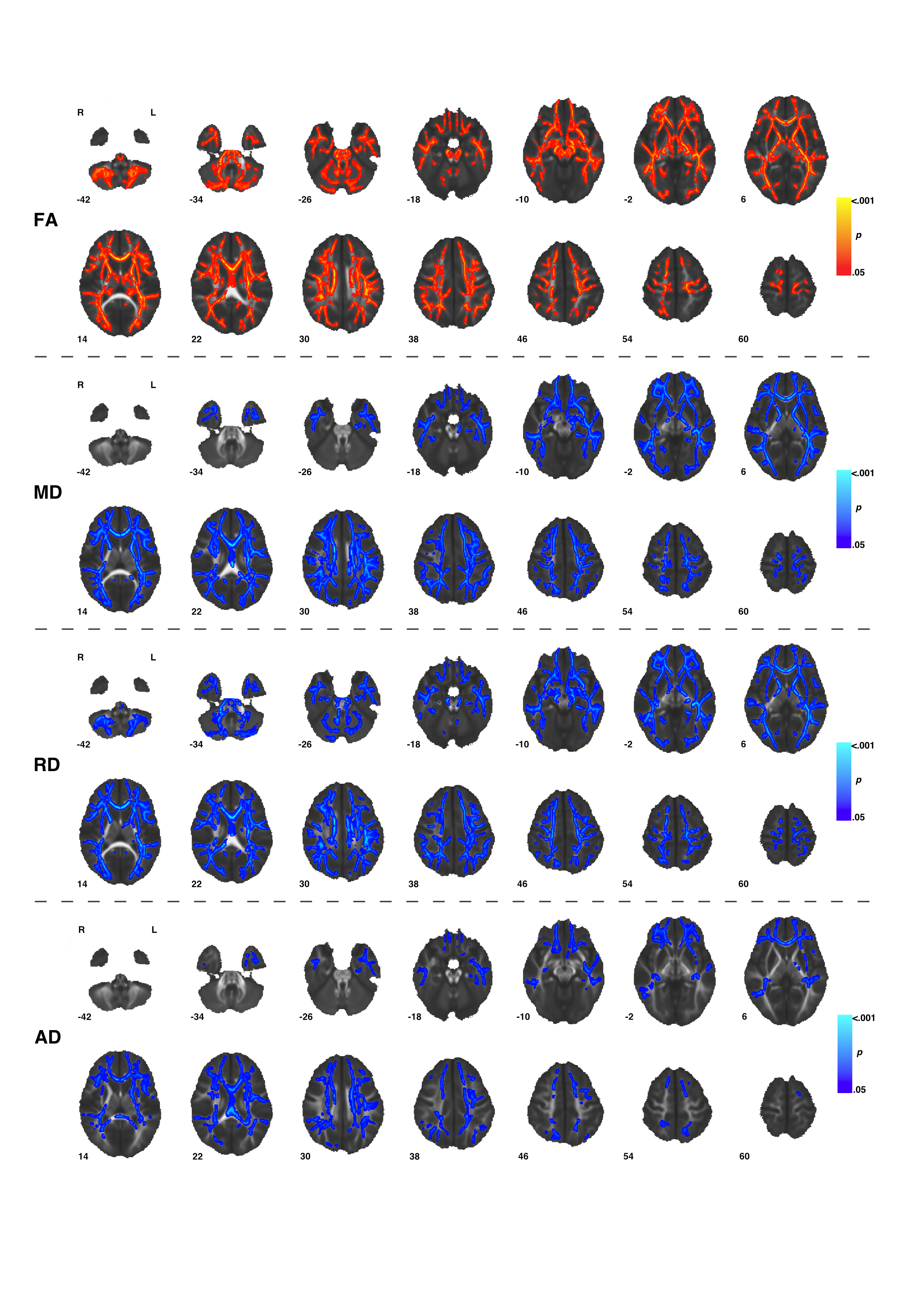

Supplement: Supplementary file 1 — Additional file 1: Figure S1. Effects of Age on Measures of Diffusion. Clusters (Additional file 3: Table S1) of significantly (TFCE p<0.05) increased fractional anisotropy (FA) and decreased mean diffusivity (MD), radial diffusivity (RD), and axial diffusivity (AD) with age are highlighted. Images are presented in R/L radiological convention with MNI z coordinates in mm. Skeletonized statistical overlays have been ‘inflated’ for display. [file 11689_2019_9291_MOESM1_ESM.tiff]

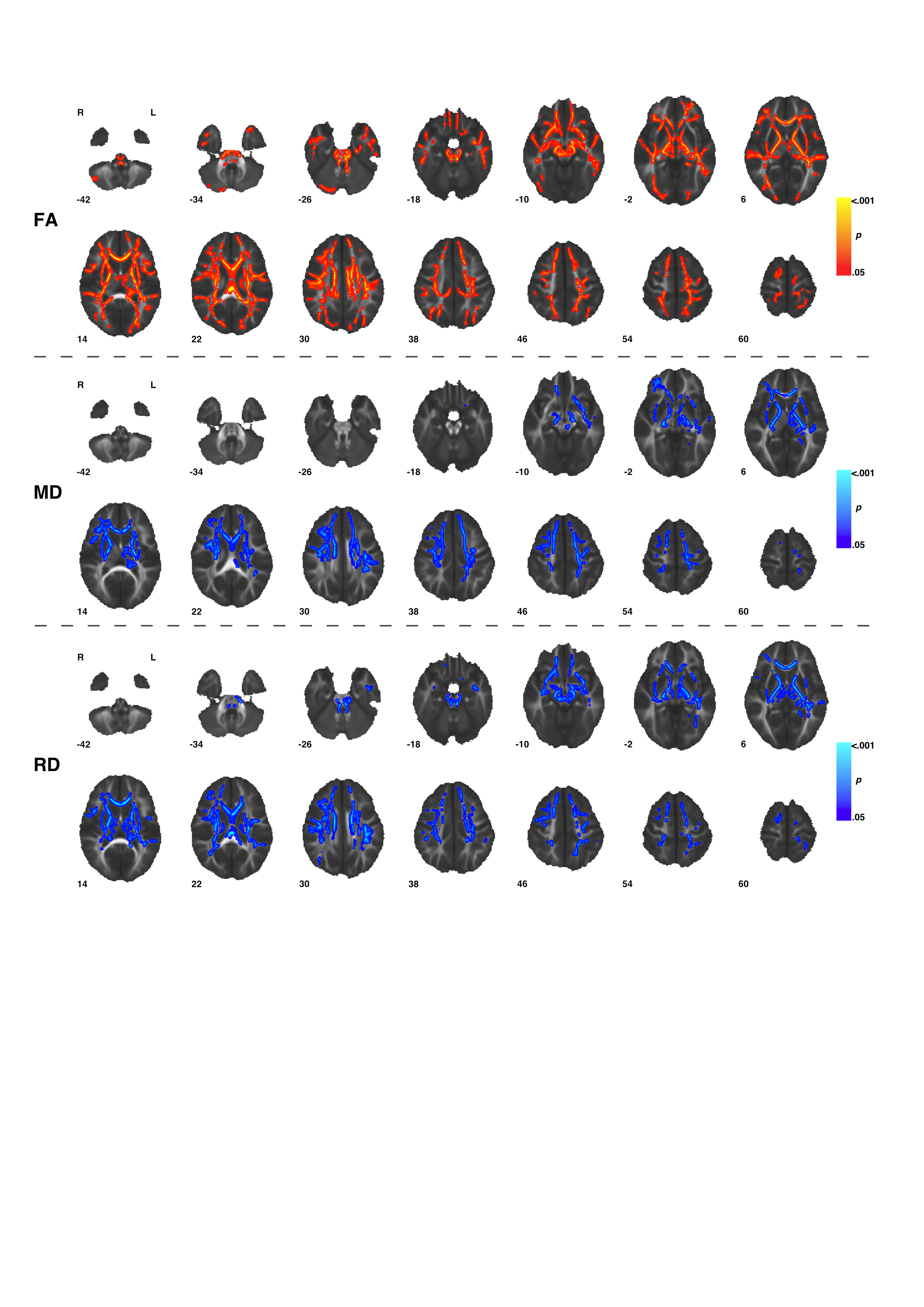

Supplement: Supplementary file 2 — Additional file 2: Figure S2. Effects of Sex on Measures of Diffusion. Clusters (Additional file 3: Table S2) showing significantly (TFCE p<0.05) increased fractional anisotropy (FA) and decreased mean diffusivity (MD) and radial diffusivity (RD) in males compared to females across diagnostic groups are highlighted. Images are presented in R/L radiological convention with MNI z coordinates in mm. Skeletonized statistical overlays have been ‘inflated’ for display. [file 11689_2019_9291_MOESM2_ESM.tiff]
