# Supplementary material for: A diffusion-weighted imaging tract-based spatial statistics study of autism spectrum disorder in preschool-aged children
Source: J Neurodev Disord. 2019 Dec 16;11:32. doi: 10.1186/s11689-019-9291-z (PMC6913008; doi:10.1186/s11689-019-9291-z)
Supplement: Supplementary file 3 — Additional file 3: Table S1. Clusters with Significant Effect of Age. Table S2. Clusters with Significant Effect of Sex. [file 11689_2019_9291_MOESM3_ESM.docx]

| **Supplementary Table 1: Clusters with Significant Effect of Age** | | | | | | | | | |
| --- | --- | --- | --- | --- | --- | --- | --- | --- | --- |
| **Effect** | **Feature** | **Cluster ID** | **Tract(s)** | ***p*** | ***t* _max_** | **voxels** | **X (mm)** | **Y (mm)** | **Z (mm)** |
|  |  |  |  |  |  |  |  |  |  |
| **Age** | **FA** | 1 | Inferior/Middle/Superior Cerebellar Peduncle, Pontine Crossing Tract, Genu/Body/Splenium Corpus Callosum, Fornix, Corticospinal Tract, Medial Lemniscus, Cerebral Peduncle, Anterior/Posterior Limbs Internal Capsule, Anterior/Superior/Posterior Corona Radiata, Posterior Thalamic Radiation, External Capsule, Cingulum, Inferior/Superior Longitudinal Fasciculus, Inferior/Superior Fronto-occipital Fasciculus, Uncinate Fasciculus | <0.001 | 6.28 | 75039 | -22 | 22 | 10 |
|  |  |  |  |  |  |  |  |  |  |
|  | **MD** | 1 | Genu/Body/Splenium Corpus Callosum, Corticospinal Tract, Cerebral Peduncle, Anterior/Posterior Limbs Internal Capsule, Anterior/Superior/Posterior Corona Radiata, Posterior Thalamic Radiation, External Capsule, Cingulum, Inferior/Superior Longitudinal Fasciculus, Inferior/Superior Fronto-occipital Fasciculus, Uncinate Fasciculus | <0.001 | -6.99 | 78949 | -46 | -20 | -18 |
|  |  |  |  |  |  |  |  |  |  |
|  | **RD** | 1 | Inferior/Middle/Superior Cerebellar Peduncle, Pontine Crossing Tract, Genu/Body/Splenium Corpus Callosum, Fornix, Corticospinal Tract, Medial Lemniscus, Cerebral Peduncle, Anterior/Posterior Limbs Internal Capsule, Anterior/Superior/Posterior Corona Radiata, Posterior Thalamic Radiation, External Capsule, Cingulum, Inferior/Superior Longitudinal Fasciculus, Inferior/Superior Fronto-occipital Fasciculus, Uncinate Fasciculus | <0.001 | -6.25 | 83287 | -21 | 24 | 8 |
|  |  |  |  |  |  |  |  |  |  |
|  | **AD** | 1 | Genu/Body/Splenium Corpus Callosum, Fornix, Medial Lemniscus, Anterior/Posterior Limbs Internal Capsule, Anterior/Superior/Posterior Corona Radiata, Posterior Thalamic Radiation, External Capsule, Cingulum, Inferior/Superior Longitudinal Fasciculus, Inferior/Superior Fronto-occipital Fasciculus, Uncinate Fasciculus | <0.001 | 4.94 | 34710 | 5 | 25 | 12 |

**Note:** Clusters of significant age effects across diffusion measures. fractional anisotropy (FA), mean diffusivity (MD), radial diffusivity (RD), axial diffusivity (AD), tracts identified according to the *MRI Atlas of Human White Matter* (Mori et al. 2005), L (left), R (right), *p* indicates the threshold free cluster enhancement corrected *p* value for the cluster, *t*_max_ indicates maximum *t* statistic within the cluster at X Y Z MNI coordinates in mm.

| **Supplementary Table 2: Clusters with Significant Effect of Sex** | | | | | | | | | |
| --- | --- | --- | --- | --- | --- | --- | --- | --- | --- |
| **Effect** | **Feature** | **Cluster ID** | **Tract(s)** | ***p*** | ***t* _max_** | **voxels** | **X (mm)** | **Y (mm)** | **Z (mm)** |
|  |  |  |  |  |  |  |  |  |  |
| **Sex** | **FA** | 1 | Middle Cerebellar Peduncle, Pontine Crossing Tract, Genu/Body/Splenium Corpus Callosum, Fornix, Corticospinal Tract, Medial Lemniscus, Inferior/Superior Cerebellar Peduncle, Cerebral Peduncle, Anterior/Posterior Limb Internal Capsule, Anterior/Superior Corona Radiata, Posterior Thalamic Radiation, External Capsule, Cingulum, Inferior/Superior Longitudinal Fasciculus, Inferior/Superior Fronto-occipital Fasciculus, Uncinate Fasciculus | <0.001 | 5.6 | 46888 | 28 | -40 | 42 |
|  |  | 2 | R Cerebellar White Matter | 0.034 | 4.25 | 374 | 26 | -81 | -26 |
|  |  | 3 | L Cingulum | 0.042 | 4.64 | 199 | -29 | -12 | -26 |
|  |  | 4 | R Cerebellar White Matter | 0.039 | 4.14 | 196 | 45 | -65 | -38 |
|  |  | 5 | L Cingulum | 0.046 | 3.38 | 79 | -25 | -40 | -4 |
|  |  | 6 | R Middle Cerebellar Peduncle | 0.049 | 4.52 | 11 | 23 | -64 | -39 |
|  |  |  |  |  |  |  |  |  |  |
|  | **MD** | 1 | Genu/Body/Splenium Corpus Callosum, R Cerebral Peduncle, R Anterior/Posterior Limb Internal Capsule, R/L Anterior/Superior/Corona Radiata, R External Capsule, R/L Superior Longitudinal Fasciculus, R Superior Fronto-Occipital Fasciculus | 0.01 | 5.21 | 17301 | 20 | -11 | 11 |
|  |  | 2 | L Cerebral Peduncle, Anterior/Posterior Limb Internal Capsule, Anterior/Superior/Posterior Corona Radiata, External Capsule, Superior Fronto-Occipital Fasciculus, Uncinate Fasciculus | 0.032 | 4.8 | 5036 | -27 | 6 | -11 |
|  |  |  |  |  |  |  |  |  |  |
|  | **RD** | 1 | Middle/Superior Cerebellar Peduncle, Pontine Crossing Tract, Genu/Body/Splenium Corpus Callosum, Fornix, Corticospinal Tract, Medial Lemniscus, Cerebral Peduncle, Anterior/Posterior Limb Internal Capsule, Anterior/Superior/Posterior Corona Radiata, Posterior Thalamic Radiation, External Capsule, Inferior/Superior Longitudinal Fasciculus, Inferior/Superior Fronto-occipital Fasciculus, Uncinate Fasciculus | 0.002 | 5.11 | 26765 | 8 | -1 | 26 |
|  |  | 2 | L Posterior Corona Radiata | 0.044 | 3.4 | 978 | -16 | -58 | 46 |
|  |  | 3 | L Posterior Thalamic Radiation | 0.047 | 3.18 | 276 | -51 | -41 | 18 |
|  |  | 4 | R Posterior Corona Radiata | 0.042 | 3.83 | 263 | 22 | -42 | 46 |
|  |  | 5 | L Posterior Corona Radiata | 0.049 | 3.07 | 33 | -20 | -43 | 31 |
|  |  | 6 | L Superior Corona Radiata | 0.049 | 2.87 | 24 | -12 | -20 | 50 |
|  |  | 7 | L Posterior Corona Radiata | 0.048 | 4.27 | 24 | -10 | -27 | 53 |
|  |  | 8 | L Posterior Corona Radiata | 0.05 | 2.96 | 10 | -27 | -28 | 58 |
|  |  | 9 | L Superior Corona Radiata | 0.05 | 2.63 | 7 | -12 | -16 | 51 |
|  |  | 10 | L Posterior Limb Internal Capsule | 0.05 | 3.54 | 4 | -3 | -4 | -13 |
|  |  | 11 | R Posterior Corona Radiata | 0.05 | 1.95 | 2 | 19 | -41 | 57 |
|  |  | 12 | R Posterior Corona Radiata | 0.05 | 1.51 | 1 | 19 | -47 | 53 |

**Note:** Clusters of significant age effects across diffusion measures. fractional anisotropy (FA), mean diffusivity (MD), radial diffusivity (RD), tracts identified according to the *MRI Atlas of Human White Matter* (Mori et al. 2005), L (left), R (right), *p* indicates the threshold free cluster enhancement corrected *p* value for the cluster, *t*_max_ indicates maximum *t* statistic within the cluster at X Y Z MNI coordinates in mm.
